# Childbirth fear in the USA during the COVID-19 pandemic: key predictors and associated birth outcomes

**DOI:** 10.1093/emph/eoad006

**Published:** 2023-04-13

**Authors:** Z M Thayer, S A Geisel-Zamora, G Uwizeye, T E Gildner

**Affiliations:** Department of Anthropology, Dartmouth College, Hanover, NH, USA; Department of Anthropology, Dartmouth College, Hanover, NH, USA; Arthur Labatt Family School of Nursing, FNB Room 2305, Arthur Labatt Family School of Nursing, University of Western Ontario, London, Canada; Department of Anthropology, Washington University in St. Louis, St. Louis, MO, USA

**Keywords:** childbirth, tokophobia, pregnancy anxiety, prenatal stress

## Abstract

**Background and objectives:**

Childbirth fear, which has been argued to have an adaptive basis, exists on a spectrum. Pathologically high levels of childbirth fear is a clinical condition called tokophobia. As a chronic stressor in pregnancy, tokophobia could impact birth outcomes. Many factors associated with tokophobia, including inadequate labor support, were exacerbated by the COVID-19 pandemic.

**Methodology:**

We used longitudinally collected data from a convenience sample of 1775 pregnant persons in the USA to evaluate the association between general and COVID-19 pandemic-related factors and tokophobia using the fear of birth scale. We also assessed associations between tokophobia, low birth weight and preterm birth when adjusting for cesarean section and other covariates among a subset of participants (*N* = 993).

**Results:**

Tokophobia was highly prevalent (62%). Mothers who self-identified as Black (odds ratio (OR) = 1.90), had lower income (OR = 1.39), had less education (OR = 1.37), had a high-risk pregnancy (OR = 1.65) or had prenatal depression (OR = 4.95) had significantly higher odds of tokophobia. Concerns about how COVID-19 could negatively affect maternal and infant health and birth experience were also associated with tokophobia (ORs from 1.51 to 1.79). Tokophobia was significantly associated with increased odds of giving birth preterm (OR = 1.93).

**Conclusions and implications:**

Tokophobia increases the odds of preterm birth and is more prevalent among individuals who are Black, have a lower income, and have less education. Tokophobia may, therefore, be an underappreciated contributor to inequities in US birth outcomes. The COVID-19 pandemic likely compounded these effects.

## BACKGROUND AND OBJECTIVES

Beginning in the 1980s, researchers in Sweden and later Finland began researching the psychological construct that would come to be defined as ‘fear of childbirth’ [1]. Childbirth fears exist on a spectrum and can relate to concerns about the ability to manage pain, the risk of harm or death to themselves or their baby, general fear and anxiety about lack of control and fear of the unknown related to childbirth.

Clinically, pathologically high levels of childbirth fear is called tokophobia. Prior diagnosis of depression or anxiety, a general fear of pain, a history of sexual abuse, nulliparity (no prior birth), and traumatic birth experiences are significant predictors of tokophobia [2]. While tokophobia is likely pathological, a moderate degree of childbirth fear may be evolutionarily adaptive if it reduces maternal and infant mortality by increasing the desire to seek assistance in labor [3].

While inconsistent measurement makes comparing childbirth fear across cultural contexts difficult, there is some evidence to suggest that cultural environment influences childbirth fear. Studies using the Wijma fear of childbirth index, a 33-item instrument that is the most used tokophobia scale, have reported a severe fear of childbirth prevalence ranging from 7.5% to 15.5% among European countries (as defined by Wijma score ≥85) [1]. In Scandinavia, tokophobia screening and clinical support are commonly offered as a standard of care, and tokophobia is lower than in other high-income settings such as Australia and the UK [4]. Notably, a systematic review reported that individuals receiving obstetric care fear childbirth more than those receiving midwifery care [5], suggesting a correlation between the type of maternity care experience and childbirth fear.

Tokophobia has not been previously evaluated in the USA using the Wijma scale. In a qualitative study, Roosevelt and Low suggested that the Wijma scale may be inappropriate for use in the US cultural context because the scale did not include all relevant sources of concern in this setting, including fear of abandonment by clinicians during labor. The only published US study utilizing an internationally validated tokophobia scale was conducted among an online convenience sample during the COVID-19 pandemic. The authors used the fear of birth scale (FOBS), a visual analog scale that has been argued to more accurately compare childbirth fear across cultural settings [4]. These authors reported a mean score of 55.65 (on a scale of 0–100), with significantly higher scores among individuals preferring an in-hospital rather than a community birth setting (i.e. birth at home or in a free-standing birth center) [7].

### Tokophobia and birth outcomes

Maternal psychosocial stress in pregnancy is a known predictor of adverse birth outcomes, including lower birth weight and preterm birth [8–11]. Therefore, it is plausible that tokophobia, which can be a substantial source of psychosocial stress during pregnancy, could also impact birth outcomes. Tokophobia has been associated with increased cesarean section delivery in both high- and low-income countries [5]. However, only two previous studies have addressed whether tokophobia predicts adverse birth outcomes independent of the impacts on cesarean section. The first was a large Finnish study that used birth registry data and surprisingly found that clinical diagnosis of tokophobia was associated with a reduced likelihood of low birth weight and preterm birth [12]. This unexpected result may relate to the fact that tokophobia is screened for in Finland, with individuals diagnosed with tokophobia then referred to additional mental health services (e.g. phobia clinics) that could improve outcomes. The second study was a longitudinal analysis of 389 pregnant people in Ireland (18 with tokophobia) that did not find a relationship between tokophobia and gestation length or birth weight. It is unclear whether this lack of association was due to no meaningful effect or was the result of limited statistical power [13].

### Current study

Since the social context of birth influences childbirth fear, the COVID-19 pandemic—which substantially affected maternity care experiences—likely impacted this measure. Notably, many of the fear loci identified in tokophobia previously, including fear of harm to self or baby, fear of poor emotional support in labor and inadequate pain management, were directly impacted by the COVID-19 pandemic. Specifically, individuals who gave birth in the early weeks and months after the pandemic was declared in the USA on 13 March 2020 were threatened with the possibility of giving birth alone, of not having access to preferred pain management strategies, and of being separated from their infant if they tested positive for SARS-CoV-2 during delivery [14, 15]. Since previous research has already demonstrated high levels of childbirth fear during the COVID-19 pandemic [7], and tokophobia is not routinely screened for or treated in the US cultural context, this provides a unique opportunity to explore how both general risk factors and pandemic-specific concerns associate with childbirth fear in the USA, as well as whether tokophobia is associated with birth outcomes.

### Study predictions

The current study assesses what factors (general and COVID-19-related) are associated with tokophobia among a sample of US-based pregnant persons, while also investigating the association between tokophobia and birth outcomes. We test three predictions:

1) Maternal health (i.e. pre-existing physical and mental health conditions, high-risk pregnancy), demographics (i.e. education, income, race/ethnicity) and provider type (obstetrician, midwife, other) will be associated with tokophobia measured during pregnancy.
2) Concerns that the COVID-19 pandemic will affect maternal and infant health and maternity care experience will be associated with tokophobia measured in pregnancy.
3) Tokophobia measured in pregnancy will be prospectively associated with low birth weight and preterm birth, independent of cesarean section delivery, high-risk pregnancy or other covariates.

## METHODOLOGY

Data come from the COVID-19 and Reproductive Effects (CARE) study, an online survey that was administered to a convenience sample recruited using informational flyers shared through social media (Facebook, Twitter, Reddit) and to US-based contacts working in maternal health [16]. Zip code data were collected to assess participant geographic distribution. Quantitative prenatal and follow-up postpartum data reported here were collected from 16 April 2020 to 4 February 2021. Complete prenatal data were available for 1775 participants. Postpartum surveys were emailed 4 weeks after the listed delivery date, with complete data available for 1113 participants. This study received ethical approval from Dartmouth College (STUDY00032045). All participants provided informed consent. The data underlying this article will be shared on reasonable request to the corresponding author.

Qualitative information was also collected; specifically, participants were asked three open-ended questions during the prenatal survey: (i) ‘What are some concerns you have regarding your pregnancy and/or giving birth? These concerns may be unrelated to COVID-19’; (ii) ‘Have these concerns changed at all due to COVID-19?’, if yes, ‘How have your concerns changed due to COVID-19?’; and (iii) ‘Is there anything else that you think would be helpful for us to understand about what it is like to be pregnant and planning to give birth during the COVID-19 pandemic?’ This information was not used in quantitative analyses but does provide contextual information presented in the study discussion.

### Independent variables

#### Prediction 1: Maternal health, demographics and provider type

Gestational age during the survey was calculated using data on the due date and date of survey completion (days used in the analysis; for summary statistics report as week or trimester); participant age (years); education (less than a bachelor’s degree, a bachelor’s degree or a degree beyond a bachelor’s degree); household income (USD) (<$49,999, $50,000–$99,999 or $100,000+); previous birth (yes/no); high-risk pregnancy (yes/no), pre-existing health conditions (yes/no), prenatal depression (estimated as Edinburgh Postnatal Depression Survey Score ≥ 15) [17] and provider type (obstetrician/gynecologist, midwife, other). Ethnicity was measured via self-report according to the Office of Management and Budget Standards. Native Hawaiian/Pacific Islander participants were re-classified as ‘other’ due to a small sample size (*N* = 3). Reference categories for categorical variables were selected according to the groups expected to be least likely to have tokophobia and are specified in the statistical analysis section.

#### Prediction 2: COVID-19-related concerns

We created an original survey to evaluate concerns specific to receiving prenatal care and giving birth during the COVID-19 pandemic (see Table 1 for full list of items). Participants responded with a 5-point Likert scale anchored on strongly disagree to strongly agree. Individuals who reported that they ‘agreed’ or ‘strongly agreed’ were categorized as ‘agree’ for that measure.

**Table 1.** Sample descriptive characteristics. Continuous variables present mean (SD) and categorical variables present N (%). Categorical variables are ordered according to prevalence.

Variable	Sample (N = 1775)
**Age**	31.3 (4.33)
**Fear of birth score**	61.2 (21.8)
**Tokophobia**	1105 (62.2%)
**Ethnicity**	
White	1541 (86.8%)
Black	109 (6.1%)
Asian	56 (3.2%)
Other	34 (1.9%)
Hispanic	23 (1.3%)
AI/AN	12 (0.7%)
**Education**	
Advanced degree	742 (41.8%)
College degree	626 (35.3%)
No college degree	407 (22.9%)
**Household Income**	
$100,000+	966 (54.4%)
$50,000-99,999	598 (33.7%)
<$49,999	211 (11.9%)
**Probable major prenatal depression (EPDS >=15)**	530 (29.9%)
**First birth**	900 (50.7%)
**High-risk pregnancy**	622 (35.0%)
**Pre-existing health conditions**	136 (7.7%)
**Primary maternity care provider**	
Obstetrician	1422 (80.1%)
Midwife	320 (18.0%)
Other provider (i.e., Family practitioner)	33 (1.9%)
**COVID-19 concerns**	
I am worried that, because of the COVID-19 crisis, I will not be able to have the people I want with me to support me during labor.	1542 (86.9%)
I am worried that if I get sick with COVID-19 my baby will be taken away from me at birth.	1534 (86.4%)
I am worried that if I get sick with COVID-19 while pregnant, it will affect my baby.	1491 (84.0%)
I am worried that if I am sick with COVID-19 during delivery I will pass the virus on to my baby.	1326 (74.7%)
I am worried that if I become sick with COVID-19 it could have long term effects on my baby.	1248 (70.3%)
I am worried that if I get sick with COVID-19 I will be treated poorly by others.	499 (28.1%)
**Birth weight (oz) ±**	120.4 (17.1)
**Low birth weight ±**	30 (3.0%)
**Gesational age (days) ±**	275.1 (9.4)
**Preterm birth±**	56 (5.6%)

± Total N = 993

#### Prediction 3: Tokophobia

FOBS was used to assess tokophobia [18] (Supplementary Figure 1). While less common than the Wijma scale, it has been validated in the USA and has been argued to be more clinically useful and easier to interpret across cultural contexts when compared to the Wijma scale [4]. This scale presents as a visual analog asking participants to use a slider to depict how they felt about their upcoming birth, with the anchors of No Fear (0) and Fear (100), and a second scale with Calm (0) and Worried (100). Cronbach’s alpha was high for the two variables in this sample (0.88). The mean score of these two questions was used to create the FOBS score. A cut-off of 54 was used to define tokophobia, consistent with prior research that found this score corresponded with the commonly used cut-off of 85 on the Wijma fear of childbirth scale [4].

### Dependent variables

#### Predictions 1 and 2

Tokophobia: (see above).

#### Prediction 3

##### Low birth weight.

Offspring birth weight (in ounces) was collected through self-report in the postnatal questionnaire. We defined low birth weight using the clinical cut-point of 5 pounds 8 ounces [19].

##### Preterm birth.

The offspring birth date reported in the postnatal questionnaire was compared to the due date reported in the antenatal questionnaire to generate gestational age at delivery (in days). We then defined preterm birth using the clinical cut-point of 259 days (37 weeks) [19].

### Statistical analysis

Data analyses and visualizations were conducted using Stata 15.1 and R [20] and RStudio [21]. Sample descriptive statistics were calculated (Table 1). To avoid influence by outliers when testing hypothesis 3, we excluded individuals who were extremely preterm (<32 weeks) or post-term (>42 weeks) (*N* = 19). We also excluded a single participant with a birth weight of 207 ounces that was more than 5 SD above the mean. All continuous variables exhibited normal distributions, with skewness values within approximately ±0.96 and kurtosis values within approximately ±4.45 [22].

Prediction 1 and 2: Unadjusted logistic regression models were conducted to evaluate if tokophobia was significantly associated with both COVID- and non-COVID-related risk factors.

Prediction 3: Unadjusted logistic regression models were initially used to test whether tokophobia was associated with low birth weight or preterm birth. To measure the association between tokophobia and birth outcomes independent of other confounders, we then ran models that adjusted for maternal ethnicity (ref: white), income (ref: highest income category), education (ref: highest education category), provider type (ref: midwife), trimester of pregnancy when the survey was completed (ref: first), prenatal depression, nulliparity, high-risk pregnancy, pre-existing health conditions, cesarean section delivery and offspring sex. Multicollinearity was not detected in either adjusted model; all VIF values were in an acceptable range of 1.02–1.56.

## RESULTS

Sample summary statistics are reported in Table 1. The mean age of the study sample was 31.3 years (SD = 4.3 years) and this was the first pregnancy for half of the study participants (50.7%). The majority of the sample self-identified as White (86.8%) and just over half (54.4%) had a household income of more than $100,000 USD/year. Thirty-five percent of participants had a high-risk pregnancy, while 18% of participants had a midwife as a primary maternity care provider. The mean FOBS score was 61.2 (SD = 21.8) with 62% of participants having FOBS > 54, indicating tokophobia. Three percent of participants gave birth to low birth weight infants, while 5.6% gave birth preterm.

### Prediction 1—Factors related to maternal health and healthcare experience will be associated with tokophobia

**Figure 1.**
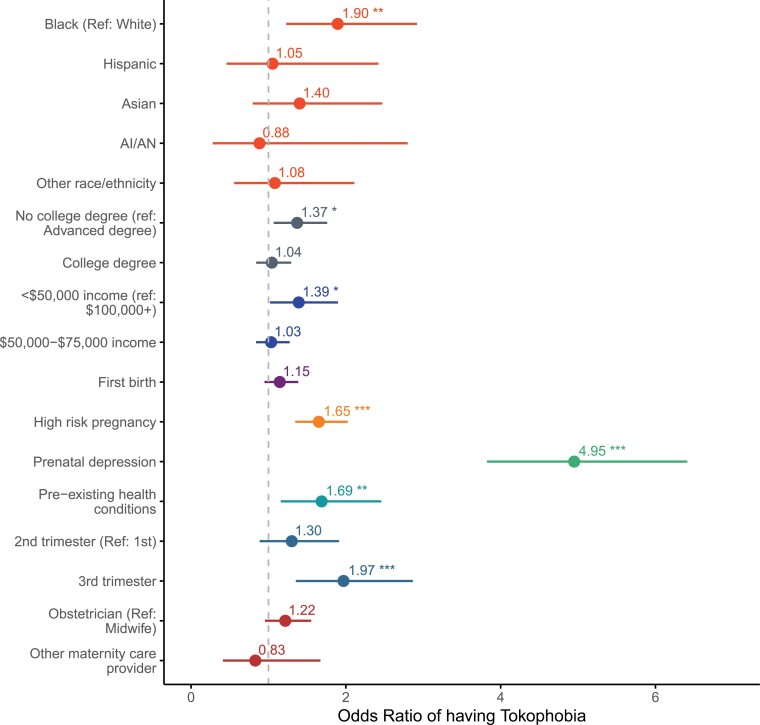
Self-reported Black ethnicity, low education, low income, high-risk pregnancy, prenatal depression, pre-existing health conditions and being in the third trimester were significantly associated with tokophobia. Reference categories for categorical variables were selected based on the group hypothesized to be least likely to have tokophobia. The figure presents the unadjusted odds ratio and 95% CI. * = *P* < 0.05; ** = *P* < 0.01; *** = *P* < 0.001.

Odds ratios and 95% confidence intervals are presented in Figure 1. Consistent with our prediction, tokophobia was significantly more common among individuals self-identifying as African American/Black (reference white: odds ratio (OR) = 1.90, 95% CI = 1.25, 2.97), those in the lowest household income category (<$50,000 per year, reference group $100,000+: OR = 1.39, 95% CI = 1.02, 1.91) and those in the lowest education category (No college degree, reference group advanced degree, OR = 1.37, 95% CI = 1.07, 1.76). In addition, having a high-risk pregnancy (OR = 1.65, 95% CI = 1.35, 2.03), a pre-existing health condition (OR = 1.69, 95% CI = 1.17, 2.48), prenatal depression (OR = 4.95, 95% CI = 3.84, 6.45) and being in the third trimester of pregnancy during the survey (relative to the first, OR = 1.97, 95% CI = 1.35, 2.89) were all associated with increased odds of tokophobia. There was a trend toward increased odds of tokophobia among participants with an obstetrician for primary care provider (reference: midwife, OR = 1.22, 95% CI = 0.95, 1.55) and for participants preparing for their first birth (reference: previous birth, OR = 1.15, 95% CI = 0.95, 1.39), but these associations were not statistically significant.

### Prediction 2—Concerns about COVID-19 pandemic-associated impacts on maternal health and maternity care experience will be associated with tokophobia

Most participants agreed or strongly agreed with a series of statements that they were concerned the COVID-19 pandemic would impact their health, their infants’ health or their maternity care experience (Table 1).

**Figure 2.**
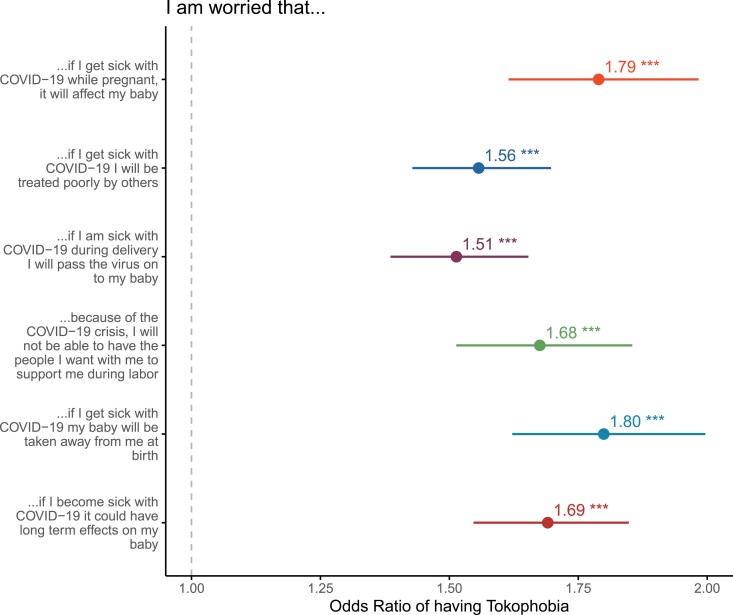
COVID-19-related concerns were significantly associated with tokophobia. The figure presents the unadjusted odds ratio and 95% CI. * = *P* < 0.05; ** = *P* < 0.01; *** = *P* < 0.001.

Consistent with our prediction, all reported concerns were significantly associated with tokophobia (Figure 2, ORs ranging from 1.51 to 1.79). Concern that COVID-19 infection would affect the baby and that the infant would be taken away at birth if the parent was positive for COVID-19 were associated with the highest odds of tokophobia (both OR = 1.79).

### Prediction 3—Tokophobia measured in pregnancy will be prospectively associated with gestational age at birth and birth weight

Consistent with our prediction, tokophobia was significantly associated with preterm birth before (OR= 1.85, 95% CI = 1.04, 3.50) and after adjusting for covariates (adjusted odds ratio (aOR) = 1.91, 95% CI = 1.02, 3.76). Individuals who had a high-risk pregnancy (aOR = 1.91, 95% CI = 1.07, 3.42) or who were in the third trimester of their pregnancy at the time of the survey (relative to first; aOR = 0.365, 95% CI = 0.14, 1.05) had significantly higher odds of delivering preterm. No other covariates were significantly associated with the odds of preterm birth.

Inconsistent with our prediction, low birth weight was not significantly associated with tokophobia in unadjusted (OR = 1.55, 95% CI = 0.72, 3.60) or adjusted (OR = 1.50, 95% CI = 0.65, 3.68) models. Cesarean section (OR = 2.25, 95% CI = 1.02, 4.93) and high-risk pregnancy (aOR = 2.83, 95% CI = 1.30, 6.48) were associated with higher odds of low birth weight birth. No other covariates were significantly associated with the odds of low birth weight.

### Conclusions and implications

Within the fear of childbirth literature, empirical research has mainly focused on fear of childbirth predictors rather than on associated outcomes. This study is the first to demonstrate a positive relationship between childbirth fear and preterm birth, finding that individuals with tokophobia have nearly twice the odds of giving birth preterm. Importantly, this association remains significant after controlling for both elective and emergency cesarean section, which previous research indicates are associated with tokophobia [23]. For context, this effect size was nearly identical to the risk of giving birth preterm for individuals diagnosed with a high-risk pregnancy.

The prevalence of tokophobia in our sample is much higher than was reported in a review of tokophobia studies in Europe, Canada, Australia and the USA, which found a childbirth fear prevalence ranging from 6% to 14% across studies [1]. However, a study in Turkey, which also used the FOBS employed in the present study, reported a median score of 65, as was observed here [24]. Our number is also slightly higher than the reported mean (56) of a similar survey among US child bearers during the COVID-19 pandemic; however, their sample only included participants anticipating a vaginal birth, and the median was not reported [7].

The high prevalence of tokophobia reported in this study is even more notable since our sample was relatively privileged, with self-identified white, highly educated participants over-represented relative to the general US birthing population. Tokophobia may, therefore, be even higher in more nationally representative samples. Additional studies are needed to understand whether the high levels of childbirth fear reported here were driven primarily by the pandemic, aspects of the US maternal health care system, high levels of pre-existing general anxiety that become elevated during pregnancy or other American cultural norms and expectations around birth.

Participants with lower income and education in this study were more likely to have tokophobia. In addition, the odds of a Black mother having tokophobia was 90% higher than the odds that a white mother did. This latter finding may reflect the unfortunate experience of obstetric racism that results in poor care delivery for many Black families in US settings [25]. Some participants explicitly stated this concern; for example, one participant wrote:

“We chose our doctor because she was POC (though not black). We wanted a black doula (but there aren’t many in our city and the ones that are present are incredibly expensive). We are deeply aware of how race plays out in care.”

-34-year-old nullipara with low-risk pregnancy

Higher childbirth fear associated with obstetric racism may therefore be a presently underappreciated contributor to the stark inequities in maternal and infant health in the USA [25–27].

#### Tokophobia influences preterm birth: Potential biological pathways

**Figure 3.**
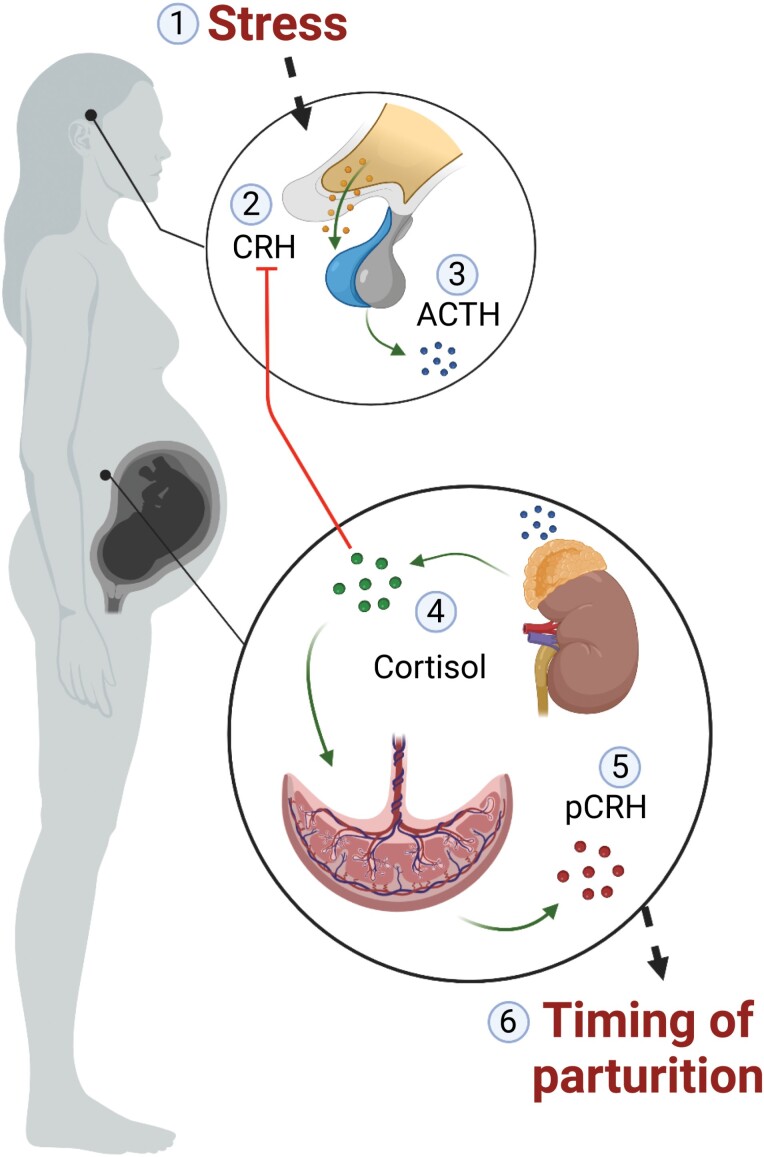
Prenatal stress triggers a hormonal cascade that has been hypothesized to impact gestation length. The HPA-axis generally operates with a negative feedback system, with cortisol production leading to a downregulation of CRH from the hypothalamus (steps 1–4). Among anthropoid primates, cortisol uniquely stimulates pCRH production from the placenta, which is believed to influence timing of labor by stimulating uterine contractions.

Our finding that tokophobia is associated with higher odds of preterm birth is consistent with other research suggesting that maternal stress experienced during pregnancy impacts gestation length [28, 29]. Prenatal stress may affect the timing of parturition through an accelerated trajectory of placentally derived corticotrophin-releasing hormone (CRH) release [30, 31] (Figure 3). Specifically, stress exposure activates the evolutionarily conserved hypothalamic–pituitary–adrenal (HPA) axis, leading to the production of hypothalamic CRH. This peptide triggers the release of adrenocorticotropic hormone (ACTH) from the pituitary, which in turn stimulates cortisol release from the adrenal cortex.

The HPA-axis is usually regulated through a negative feedback loop, with cortisol decreasing sensitivity to CRH in the hypothalamus and reducing subsequent ACTH and cortisol release. However, during pregnancy, cortisol stimulates the production of placental CRH, leading to a positive feedback loop of pCRH, ACTH, and cortisol. These peptides/hormones increase exponentially across gestation and play multiple vital roles, including facilitating fetal maturation and influencing the timing of spontaneous labor [32–34]. Elevated CRH levels and steeper increases in pCRH are associated with preterm birth [34]. Tokophobia may, therefore, impact gestation length through influences on the trajectory of pCRH increase across gestation.

#### COVID-19 has exacerbated childbirth fear

The COVID-19 pandemic exacerbated existing weaknesses in the US maternity care system [35], potentially contributing to high tokophobia in the present sample. Rapidly changing hospital regulations following the announcement of the pandemic in the USA limited the number of support persons allowed in the delivery room while also leading parents to fear or even experience separation from their newborns after testing positive for COVID-19 [14]. Furthermore, our study participants expressed concern that hospital-based care may increase COVID-19 infection risk. A critical aspect of the COVID-19 pandemic is, therefore, that it overlapped with and amplified many pre-existing factors leading to childbirth fear, including fear of unwanted medical intervention, lack of support, and risk of harm to self or child. As an example, one participant wrote:

“I’m terrified all the way around as it is my first pregnancy. You name it, I’ve worried about it. The COVID-19 stuff just adds more fear.”

-30-year-old nullipara with low-risk pregnancy

One of the specific pathways through which the COVID-19 pandemic has exacerbated childbirth fear is the threat many participants experienced of having to give birth alone. One participant wrote that she was:

“…afraid I’d end up with a c-section due to my lack of support from a doula, midwives, or other friends.”

-39-year-old multipara with high-risk pregnancy

Assisted childbirth is considered a human universal and is regarded as an important component of our evolutionary history [3, 36]. There is strong empirical evidence that emotional support is essential for positive birth outcomes; a Cochrane review found that continuous birth support was positively associated with odds of spontaneous vaginal birth (i.e. without forceps or cesarean) [37]. Thus, pandemic-associated changes to the availability of emotional support during labor clearly impacted participant childbirth fear.

#### Evolutionary perspectives on childbirth fear and gestation length

Childbirth is an essential component of human reproduction. And yet, many individuals experience severe childbirth fear, clinically defined as tokophobia. We can imagine at least two plausible evolutionary hypotheses that could help explain why pathologically high rates of childbirth fear occur. One explanation is that tokophobia reflects the development of an extreme version of an adaptive response. Specifically, a modest amount of childbirth fear may be adaptive if it promotes a desire for assistance in labor and delivery, thereby reducing maternal and infant mortality [3]. Since childbirth fear exists on a spectrum, pathologically high levels of childbirth fear may develop in a subset of individuals at the high end of the childbirth fear distribution. However, our findings show that tokophobia increases the odds of preterm birth, which could negatively affect offspring survival. Maternal depression and anxiety resulting from tokophobia could also negatively impact longer-term maternal and child well-being. Our results, therefore, do not support this hypothesis, but instead suggest that tokophobia would likely be selected against given probable negative health and fitness effects, as documented here.

A second, and more likely, hypothesis for the presence of tokophobia in cultural contexts like the USA is that it reflects a mismatch between the birth settings we likely experienced for much of our evolutionary history (i.e. familiar environments with known care attendants) and those where many individuals currently give birth (i.e. unfamiliar birth facilities with unknown care attendants) [3, 38]. The mismatch may also occur at the level of knowledge and expectations about birth since the shift in birth location and the medicalization of childbirth has influenced where and how knowledge about birth is learned (i.e. from medical professionals versus maternal kin and social networks) [38, 39]. This mismatch is likely most significant for socially disadvantaged individuals, who are more likely to experience bias and mistreatment in maternity care settings [40]. A prediction of this hypothesis is that maternity care settings characterized by safety and trust will be associated with reduced tokophobia. Consistent with this hypothesis, the community-birth setting has been associated with lower childbirth fear [7, 41]. In addition, a post-hoc analysis in our sample demonstrated a significant inverse association between satisfaction with provider and childbirth fear (*r* = −0.20, *P* < 0.0001; see [42] for description of provider satisfaction measure). Importantly, if tokophobia is indeed the result of an evolutionary mismatch scenario, it is unlikely to be adaptive.

Notably, full-term pregnancy in humans (280 days, or 40 weeks) is defined because 39–40 weeks is the most common gestation length and the gestation length associated with the lowest incidence of neonatal mortality [43]. This coupling suggests that there has likely been a strong selection of the genetic and physiological mechanisms that determine gestation length [43].

That said, shorter gestation, including preterm birth, may not be a maladaptive response in all instances; for example, Pike argued that shorter gestation length may be adaptive if it increases maternal or offspring survival in the face of compromised maternal or environmental conditions (e.g. maternal infection, undernutrition or psychosocial stress) [44]. Yet, while potentially beneficial in some instances for ensuring maternal longevity or offspring survival, early parturition can also result in tradeoffs that compromise an offspring’s long-term health. This hypothesis suggests that there is plasticity in human gestation length, with tradeoffs between current and future conditions potentially affecting the optimal timing of parturition. If true, this evolved sensitivity likely emerged in response to fundamentally different types of ecological and psychosocial stressors than those experienced in contemporary society. This means that the response of earlier parturition following psychosocial stress from obstetric racism, for example, may reflect an evolutionary mismatch.

### Limitations

Our sample consists of a disproportionally high number of participants who self-identify as white, are highly educated, and are from high-income households relative to the general US birthing population. As indicated by our findings, tokophobia is higher among Black mothers, likely due to racism and other inequities in maternity care experienced by this population [25, 40]. Additionally, these fears may have been exacerbated during the pandemic due to the disproportionate risk of infection and severe COVID-19 symptoms experienced by communities of color [45]. More research is needed on nationally representative samples to understand the true prevalence of tokophobia within the general population, and how this prevalence varies according to participant demographics and COVID-19-related factors.

Recent research has highlighted the strengths of the FOBS in both clinical settings and application across cultural contexts, mainly since there are issues of translation of several instrument items of the Wijma scale into English [4]. A qualitative study in the USA also questioned the utility of the Wijma scale measure for this population due to the lack of inclusion of questions about factors strongly associated with childbirth fear in this context, including provider mistreatment [6]. That said, the Wijma scale is considered by many to be the gold standard for fear of childbirth research. It would be helpful to conduct a study in the USA comparing the general utility of the Wijma to the FOBS, as has been done in Australia [4].

Consistent with other online survey samples conducted during the COVID-19 pandemic, we had substantial attrition between the first and second surveys [46]. Specifically, 31% of participants who consented to be re-contacted after the prenatal survey did not participate in the postnatal survey. Individuals with tokophobia were significantly more likely to be lost to follow-up (*P* = 0.04); however, the difference was slight (62% in overall sample with tokophobia vs. 60% in follow-up sample). Nonetheless, the loss to follow-up among individuals with higher childbirth fear has likely biased our results towards the null since we would predict that higher childbirth fear scores would be more strongly associated with adverse birth outcomes.

### Future directions

While this analysis has focused on the impacts of tokophobia on birth outcomes, tokophobia has consequences beyond this, including on the process of childbirth and the postpartum experience. For example, tokophobia is associated with prolonged labor, and the use of epidural and obstetric complications [47]. Additional research is needed to understand the impacts of childbirth fear on outcomes across the perinatal period, particularly in more ethnically and socioeconomically diverse samples.

## CONCLUSIONS

Research on tokophobia originated in Scandinavia, with most related studies occurring in this cultural context. The only previous study to find a significant relationship between tokophobia and birth outcomes surprisingly reported that higher childbirth fear was associated with lower adverse birth outcomes [12]. This finding may reflect that this study was conducted in Finland, where tokophobia is screened and treated as the standard of care, potentially resulting in improved outcomes. In contrast, our analysis of US mothers during the COVID-19 pandemic demonstrated a very high prevalence of tokophobia. Notably, tokophobia is neither screened nor treated as the standard of care in the US maternity system. We also found that tokophobia was associated with significantly higher odds of giving birth preterm, potentially due to higher anxiety and altered hormone profiles that influence the timing of spontaneous labor.

It is important to distinguish between moderate childbirth fear, which may have an adaptive basis, and tokophobia, which is considered a pathologically high level of fear. Low levels of fear could be adaptive if it motivates individuals to seek care and assistance in labor [3]. However, the high prevalence of tokophobia reported here may reflect an evolutionary mismatch, with contemporary birth environments being characterized by a lack of familiar spaces and individuals, and disruptions to familial and peer transmission of birth knowledge. This mismatch may be particularly apparent for groups that experience greater mistrust and abuse from obstetric providers [40, 48, 49].

Since tokophobia is more prevalent among individuals who are Black, lower income and who have less education, and tokophobia increases the odds of preterm birth, our results suggest that tokophobia may be an additional and presently underappreciated contributor to ethnic and socioeconomic inequities in birth outcomes in the USA. Cumulatively, these results demonstrate the need to include tokophobia as a measure in future maternal health studies in the USA and elsewhere. Professional medical associations should also recommend screening and treatment for tokophobia, since treatment has been shown to decrease fear and improve maternal self-efficacy [13, 50]. Finally, future work on tokophobia should consider how ultimate (evolutionary) and proximate (cultural, developmental) factors jointly shape birth expectations and childbirth fear.

## Supplementary Material

eoad006_suppl_Supplementary_File
