# Supplementary material for: Childbirth fear in the USA during the COVID-19 pandemic: key predictors and associated birth outcomes
Source: Evol Med Public Health. 2023 Apr 13;11(1):101–11. doi: 10.1093/emph/eoad006 (PMC10114526; doi:10.1093/emph/eoad006)

**Supplementary File 1**

**Fear of Birth Scale Instrument**

How do you feel right now about the approaching birth? Please mark with the slider on the lines below.


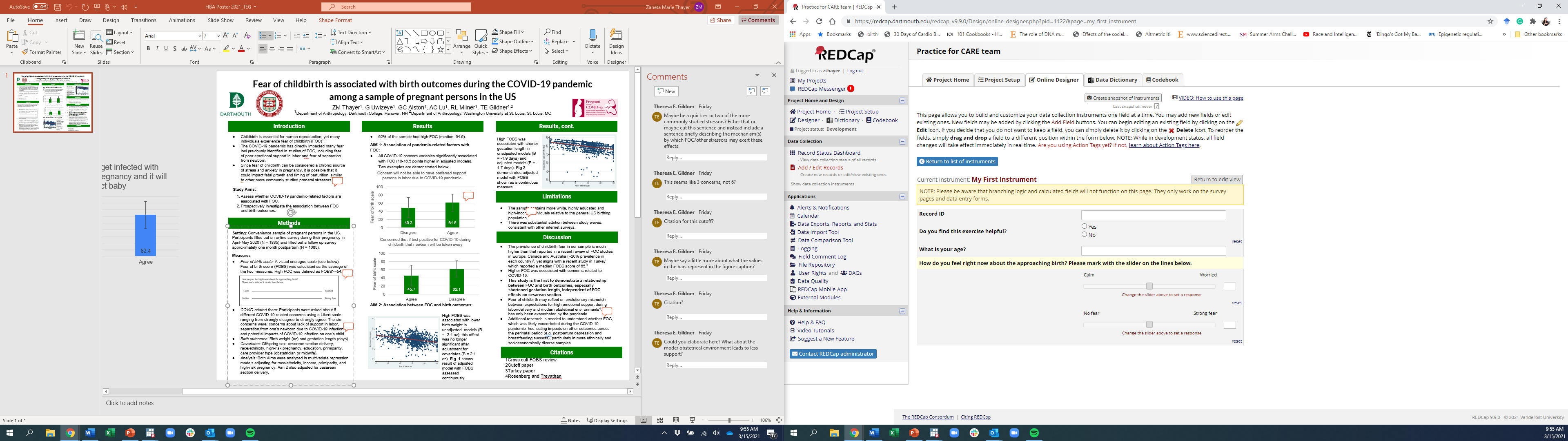

Supplement: eoad006_suppl_Supplementary_File [file eoad006_suppl_supplementary_file.docx]
